# Potential Smoothened Inhibitor from Traditional Chinese Medicine against the Disease of Diabetes, Obesity, and Cancer

**DOI:** 10.1155/2014/873010

**Published:** 2014-07-01

**Authors:** Kuan-Chung Chen, Mao-Feng Sun, Hsin-Yi Chen, Cheng-Chun Lee, Calvin Yu-Chian Chen

**Affiliations:** ^1^School of Pharmacy, China Medical University, Taichung 40402, Taiwan; ^2^School of Chinese Medicine, College of Chinese Medicine, China Medical University, Taichung 40402, Taiwan; ^3^Department of Acupuncture, China Medical University Hospital, Taichung, Taiwan; ^4^Research Center for Chinese Medicine & Acupuncture, China Medical University, Taichung, Taiwan; ^5^Department of Biomedical Informatics, Asia University, Taichung 41354, Taiwan; ^6^School of Medicine, College of Medicine, China Medical University, Taichung 40402, Taiwan; ^7^Human Genetic Center, Department of Medical Research, China Medical University Hospital, Taichung, Taiwan

## Abstract

Nowadays, obesity becomes a serious global problem, which can induce a series of diseases such as type 2 diabetes mellitus, cancer, cardiovascular disease, metabolic syndrome, and stoke. For the mechanisms of diseases, the hedgehog signaling pathway plays an important role in body patterning during embryogenesis. For this reason, smoothened homologue (Smo) protein had been indicated as the drug target. In addition, the small-molecule Smo inhibitor had also been used in oncology clinical trials. To improve drug development of TCM compounds, we aim to investigate the potent lead compounds as Smo inhibitor from the TCM compounds in TCM Database@Taiwan. The top three TCM compounds, precatorine, labiatic acid, and 2,2′-[benzene-1,4-diylbis(methanediyloxybenzene-4,1-diyl)]bis(oxoacetic acid), have displayed higher potent binding affinities than the positive control, LY2940680, in the docking simulation. After MD simulations, which can optimize the result of docking simulation and validate the stability of H-bonds between each ligand and Smo protein under dynamic conditions, top three TCM compounds maintain most of interactions with Smo protein, which keep the ligand binding stable in the binding domain. Hence, we propose precatorine, labiatic acid, and 2,2′-[benzene-1,4-diylbis(methanediyloxybenzene-4,1-diyl)]bis(oxoacetic acid) as potential lead compounds for further study in drug development process with the Smo protein.

## 1. Introduction

Nowadays, obesity, which is caused by the body's inability to handle excessive energy intake, becomes a serious global problem. It can induce a series of diseases such as type 2 diabetes mellitus, cancer, cardiovascular disease, metabolic syndrome, and stroke [1, 2]. In fact, the diseases of diabetes, obesity, and cancer have the dysregulated intracellular signaling and altered metabolic state [3]. Nowadays, increasing numbers of distinct mechanisms of diseases have been determined [4–6]. According to these mechanisms, increasing numbers of potential target proteins for drug design against each disease have been identified [7, 8]. The hedgehog signaling pathway plays an important role in body patterning during embryogenesis [9]. Abnormalities in hedgehog signaling pathway can lead to diabetes, obesity, and cancer [10–14]. As hedgehog pathway genes encoding patched homologue 1 (Ptch1) and smoothened homologue (Smo), Smo protein had been indicated as the drug target, and the small-molecule Smo inhibitor had been used in oncology clinical trials [15–18].

Many* in silico* researches had indicated that compounds extracted from traditional Chinese medicine (TCM) can be used as potential lead compounds for many different diseases [19], such as cancer [20–23], diabetes [24], inflammation [25], influenza [26], metabolic syndrome [27, 28], stroke [29–32], viral infection [33], and some other diseases [34, 35]. To improve drug development of TCM compounds, we aim to investigate the potent lead compounds as Smo inhibitor from the TCM compounds in TCM Database@Taiwan [36]. As structural disordered residues in the protein may lead to the side effect and influence the ligand to bind with target protein [37, 38], the disordered residues of Smo protein were predicted before virtual screening. After virtual screening of the TCM compounds, as the interactions between protein and ligand in the docking simulation may not be stable under dynamic conditions, the molecular dynamics (MD) simulations were performed to validate the stability of those interactions.

## 2. Materials and Methods

### 2.1. Data Collection

The X-ray crystallography structure of the human smoothened receptor (Smo) was obtained from RCSB Protein Data Bank with PDB ID: 4JKV [39]. PONDR-Fit [40] protocol was employed to predict the disordered amino acids for the sequence of Smo protein from Swiss-Prot (UniProtKB: Q99835). For the protein preparation, Prepare Protein module in Discovery Studio 2.5 (DS2.5) was employed to protonate the final structure of protein with Chemistry at HARvard Macromolecular Mechanics (CHARMM) force field [41] and remove crystal water. The binding site for virtual screening was defined by the volume of the cocrystallized antitumor agent, LY2940680. Prepare Ligand module in DS2.5 was employed to protonate the final structure of TCM compounds from TCM Database@Taiwan [36], and Lipinski's Rule of Five [42] was applied to filter the TCM compounds after virtual screening.

### 2.2. Docking Simulation

LigandFit protocol [43] in DS 2.5 was employed to virtually screen the TCM compounds by docking ligands into the binding site using a shape filter and Monte-Carlo ligand conformation generation. The result of docking was then optionally minimized with CHARMM force field [41] and evaluated with Dock Score energy function as follows:

(1) Dock  Score=−(ligand receptor interaction energy   +  ligand internal energy).

The clustering of saved docking pose was performed to reject the similar poses.

### 2.3. Molecular Dynamics (MD) Simulation

Gromacs [44] was employed to simulate each protein-ligand complex under dynamic conditions using classical molecular dynamics theory. The pdb2gmx protocol of Gromacs and SwissParam program [45] were employed to provide topology and parameters for Smo protein with charmm27 force field and each ligand, respectively. The Gromacs program sets the dimensions of the cubic box based upon setting the box edge approx 12 Å from the molecules periphery and solvated using TIP3P water model. Steepest descent [46] is one of the common algorithms for minimization. For this algorithm, new positions are calculated by the equation as follows:

(2) rn+1=rn+Fnmax⁡(|Fn|)hnIf  Vn+1<Vn,  rn+1  accepted  and  hn+1=1.2hnotherwise,rn+1  rejected  and  hn=0.2hn,

where *r* is the vector of all 3N coordinates, *h*_*n*_ is the maximum displacement and initial *h*_0_ is given in unit of 0.01 nm, and *F*_*n*_ is the force or the negative gradient of the potential *V*.

The algorithm stops when max⁡(|*F*_*n*_|) < *ε* or complete the maximum number of iterations defined in the protocol. After a steepest descent minimization with a maximum of 5,000 steps was employed to remove bad van der Waals contacts, it created a neutral system using 0.145 M NaCl model. Then another steepest descent minimization with a maximum of 5,000 steps was employed to remove bad van der Waals contacts. For the equilibration, the position-restrained molecular dynamics with the Linear Constraint algorithm for all bonds was performed with NVT equilibration, Berendsen weak thermal coupling method, and Particle Mesh Ewald method. The Berendsen weak thermal coupling method mimics with first-order kinetics an external heat bath with given temperature 300 K and slowly corrected the temperature deviation of the system by the equation as follows:

(3) $$\begin{array}{c} \frac{\partial T}{\partial t}=\frac{T_{0}-T}{\tau }, \end{array}$$

where *T*_0_ is given temperature 300 K and *τ* is a time constant in unit of 0.1 ps.

The MD program was then employed to simulate a total of 5000 ps production simulation with time step in unit of 2 fs under Particle Mesh Ewald (PME) option and NPT ensembles. A series of protocols in Gromacs was employed to analyze the MD trajectories.

## 3. Results and Discussion

### 3.1. Disordered Protein Prediction

**Figure 1:**
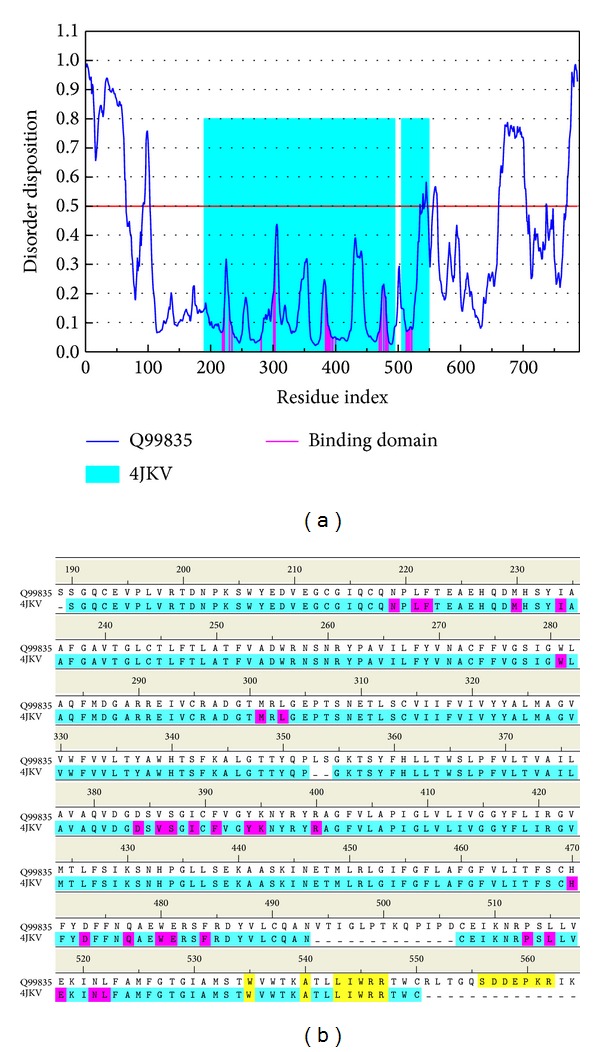
Disordered disposition predicted by PONDR-Fit. Sequence alignment with disordered residues (yellow regions) and residues in the binding domain (magenta regions).

The disordered disposition for the sequence of Smo protein from Swiss-Prot (UniProtKB: Q99835) predicted by PONDR-Fit was illustrated in Figure 1. As the residues in the binding domain do not lie in the disordered region, the binding domain of Smo protein has a stable structure in protein folding.

### 3.2. Docking Simulation

**Figure 2:**
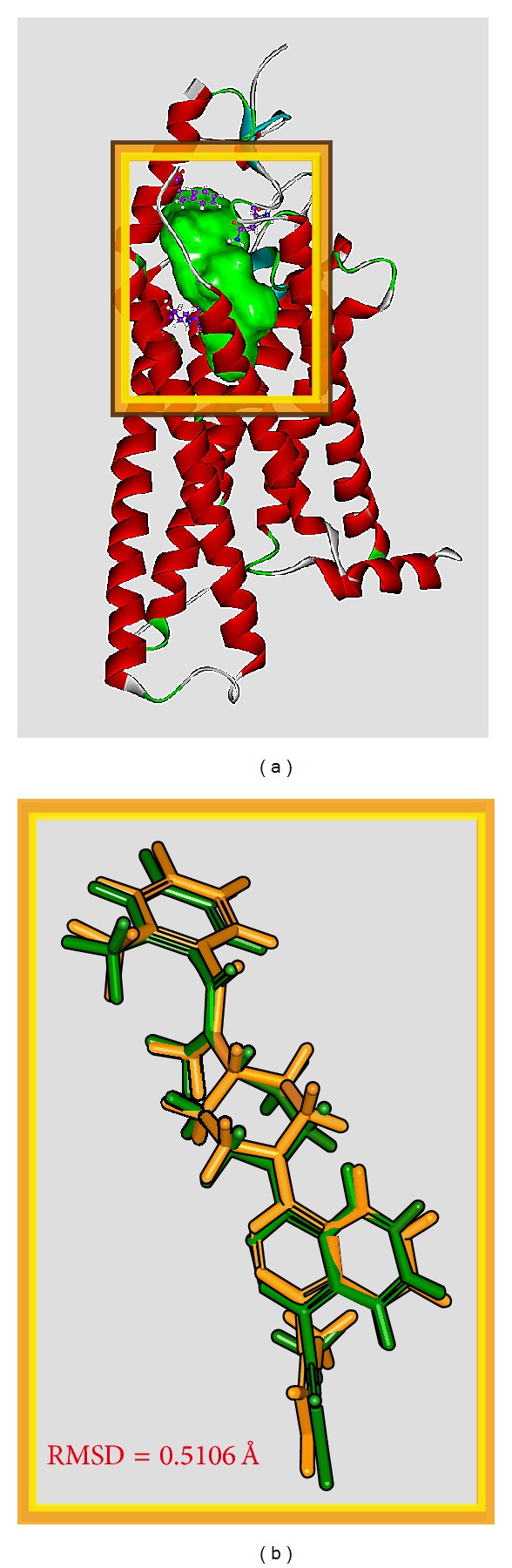
(a) Binding site of Smo protein defined as the volume of LY2940680. (b) Root-mean-square deviation value between crystallized structure (orange) and docking pose (green) of LY2940680.

**Figure 3:**
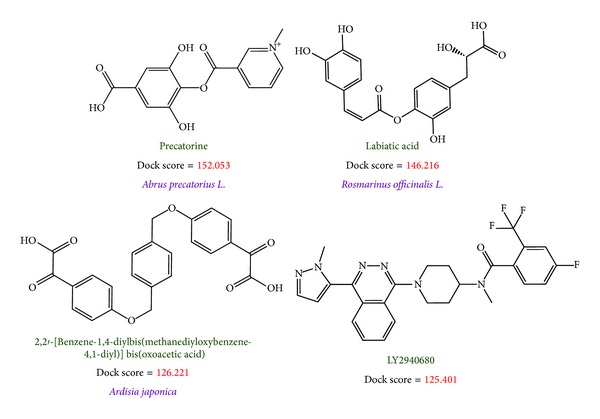
Chemical scaffold of controls and top three TCM candidates with their scoring function and sources.

**Figure 4:**
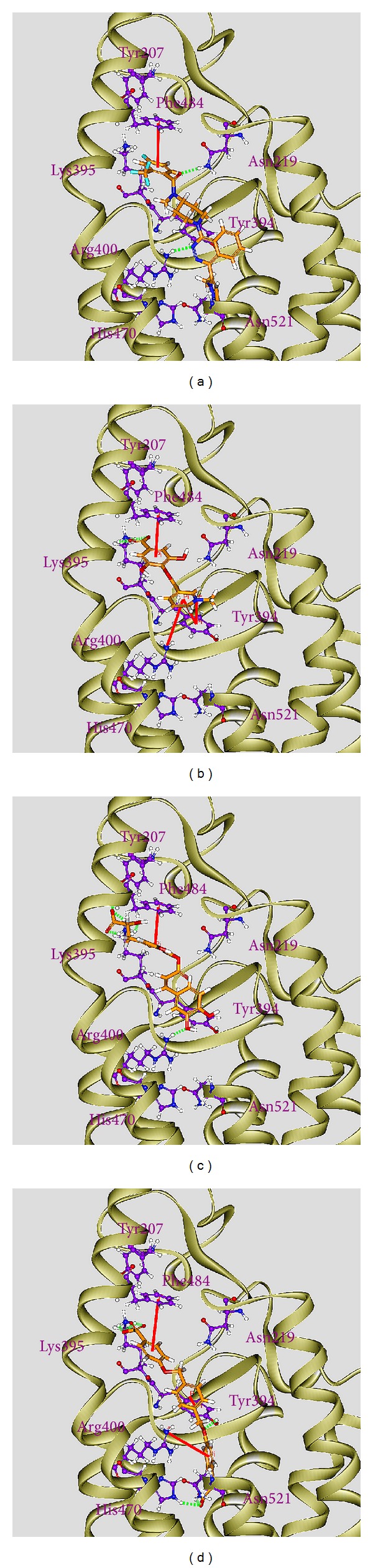
Docking pose of Smo protein complexes with (a) LY2940680, (b) precatorine, (c) labiatic acid, and (d) 2,2′-[benzene-1,4-diylbis(methanediyloxybenzene-4,1-diyl)]bis(oxoacetic acid).

Before virtual screening, the cocrystallized antitumor agent, LY2940680, had been redocked by LigandFit protocol into the binding site defined by the volume of LY2940680 (Figure 2(a)) to validate the accuracy of LigandFit protocol. The Root-mean-square deviation value between crystallized structure and docking pose of LY2940680 is 0.5106 Å (Figure 2(b)). After virtual screening, the chemical scaffold top TCM compounds ranked by Dock Score [43] and LY2940680 are illustrated in Figure 3. The scoring function of Dock Score indicates that the top three TCM compounds have higher binding affinities than LY2940680. The top three TCM compounds, precatorine, labiatic acid, and 2,2′-[benzene-1,4-diylbis(methanediyloxybenzene-4,1-diyl)]bis(oxoacetic acid), are extracted from* Abrus precatorius* L.,* Rosmarinus officinalis* L., and* Ardisia japonica*, respectively. According to the docking poses in Figure 4, for positive control, LY2940680, there exists a *π* interaction with residue Phe484 and hydrogen bonds (H-bonds) with residues Asn219 and Arg400. Precatorine has *π* interactions with residues Tyr394, Arg400, Phe484, and H-bonds with residue Lys395. Labiatic acid has a *π* interaction with residue Phe484 and H-bonds with residues Tyr207, Lys395, and Arg400. The top 3 compounds have *π* interactions with residues Tyr394, Arg400, Phe484, and H-bonds with resides Tyr394, Lys395, His470, and Asn521. The docking poses displayed in Figure 4 indicate that each compound has a *π* interaction with residue Phe484 and interaction with common residues Lys395 and Arg400. Those interactions stabilize each compound in the binding domain of Smo protein.

### 3.3. Molecular Dynamics Simulation

**Figure 5:**
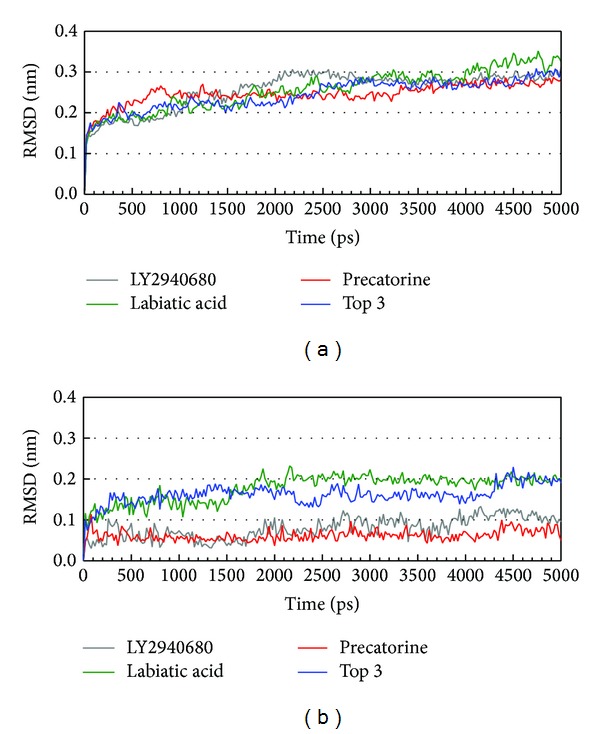
Root-mean-square deviations in units of nm for protein (a) and ligand (b) over 5000 ps of MD simulation in Smo protein complexes with LY2940680, precatorine, labiatic acid, and 2,2′-[benzene-1,4-diylbis(methanediyloxybenzene-4,1-diyl)]bis(oxoacetic acid).

**Figure 6:**
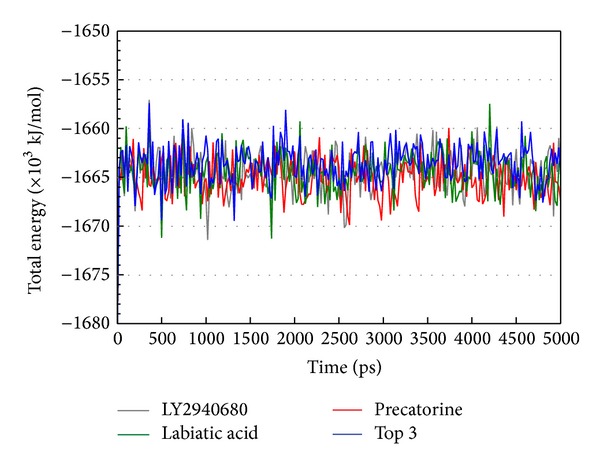
Total energy for complex over 5000 ps of MD simulation in Smo protein complexes with LY2940680, precatorine, labiatic acid, and 2,2′-[benzene-1,4-diylbis(methanediyloxybenzene-4,1-diyl)]bis(oxoacetic acid).

**Figure 7:**
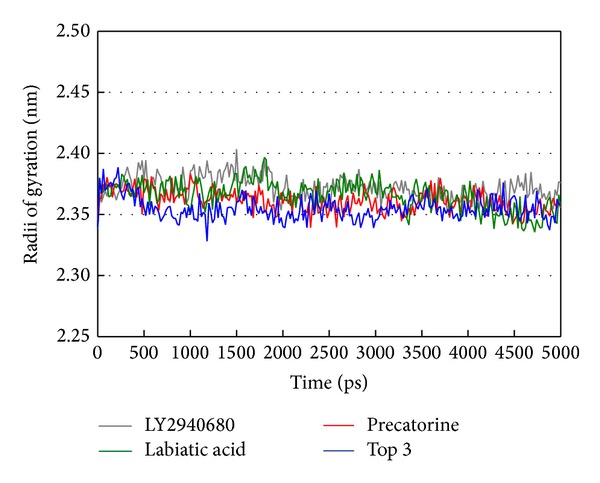
Radii of gyration for protein over 5000 ps of MD simulation in Smo protein complexes with LY2940680, precatorine, labiatic acid, and 2,2′-[benzene-1,4-diylbis(methanediyloxybenzene-4,1-diyl)]bis(oxoacetic acid).

**Figure 8:**
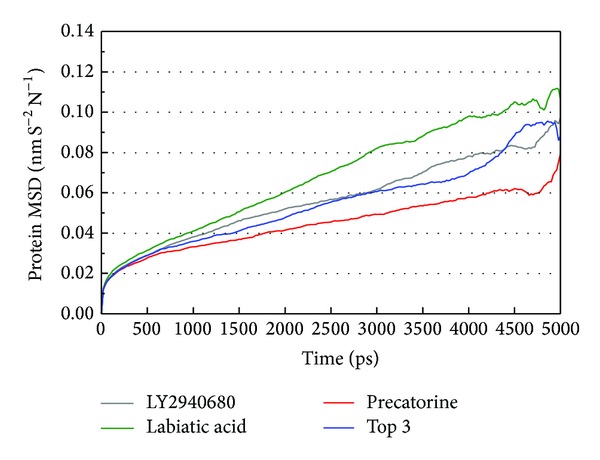
Mean square displacement (MSD) for protein over 5000 ps of MD simulation in Smo protein complexes with LY2940680, precatorine, labiatic acid, and 2,2′-[benzene-1,4-diylbis(methanediyloxybenzene-4,1-diyl)]bis(oxoacetic acid).

**Figure 9:**
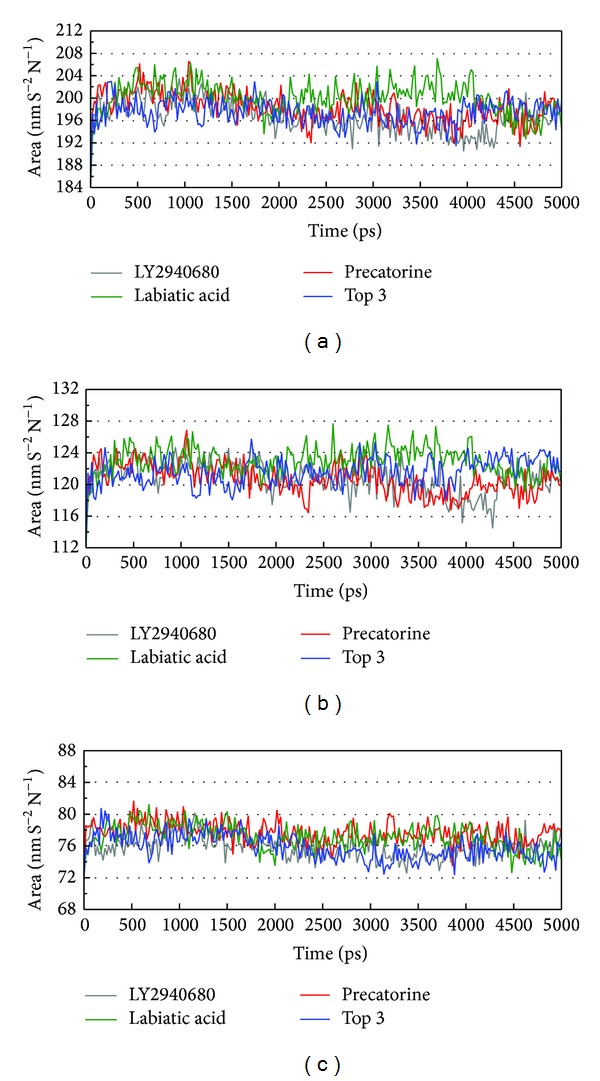
Variation of (a) total solvent accessible surface area, (b) hydrophobic surface area, and (c) hydrophilic surface area over 5000 ps of MD simulation for Smo protein complexes with LY2940680, precatorine, labiatic acid, and 2,2′-[benzene-1,4-diylbis(methanediyloxybenzene-4,1-diyl)]bis(oxoacetic acid).

**Figure 10:**
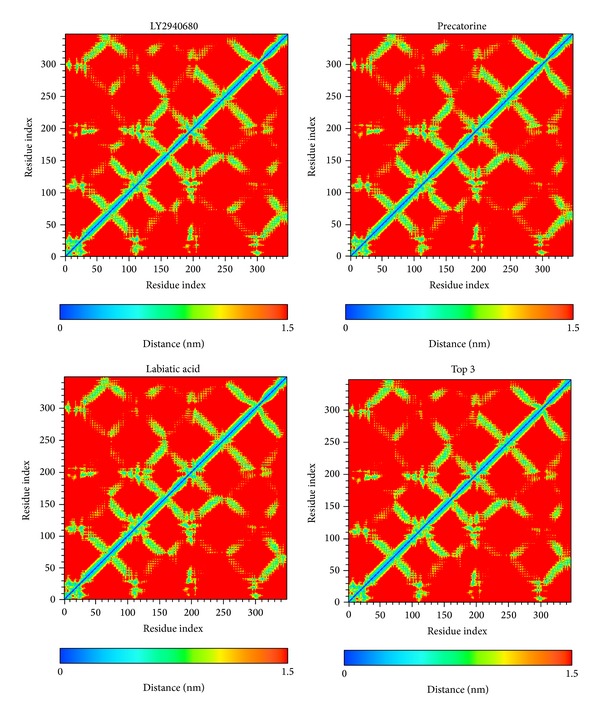
Smallest distance between residue pairs for protein over 5000 ps of MD simulation in Smo protein complexes with LY2940680, precatorine, labiatic acid, and 2,2′-[benzene-1,4-diylbis(methanediyloxybenzene-4,1-diyl)]bis(oxoacetic acid).

The docking simulation performed by LigandFit protocol docks compounds into binding site using a shape-based docking. Although the Monte-Carlo techniques had been employed to simulate the flexible compound by generating sets of compound conformations, the structure of target protein is a rigid body of Smo protein from the crystal structure. As the interactions between protein and ligand in the docking simulation may not be stable under dynamic conditions, the molecular dynamics (MD) simulations were performed to validate the stability of those interactions. The root-mean-square deviations (RMSDs) for each protein and ligand were displayed in Figure 5. They indicate the atomic fluctuations during MD simulation for each protein and ligand. Figure 5 shows that the atomic fluctuations of each complex tend to be stable after 4700 ps of MD simulation. The variations of total energy for each complex during 5000 ps of MD simulation were illustrated in Figure 6, which indicate that Smo protein docking with the top three TCM compounds has similar variation of total energy, and there is no significant change of total energy for each complex during 5000 ps of MD simulation. The variation of radius of gyration and mean square displacement (MSD) for proteins in each complex during 5000 ps of MD simulation was illustrated in Figures 7 and 8, respectively. They indicate that Smo protein docking with the top three TCM compounds has similar compactness and diffusion constant under dynamic conditions as LY2940680. The variation of solvent accessible surface area in Figure 9 can also be used to discuss the effect of each ligand for the Smo protein after docking. In Figure 9, it can be seen clearly that the Smo protein in each complex has similar solvent accessible surface area when the RMSDs tend to be stable after 4700 ps of MD simulation. The smallest distance between residue pairs for Smo protein in each complex illustrated in Figure 10 also has similar distance matrices. They indicate that the top three TCM compounds may cause similar influence for Smo protein as LY2940680.

**Figure 11:**
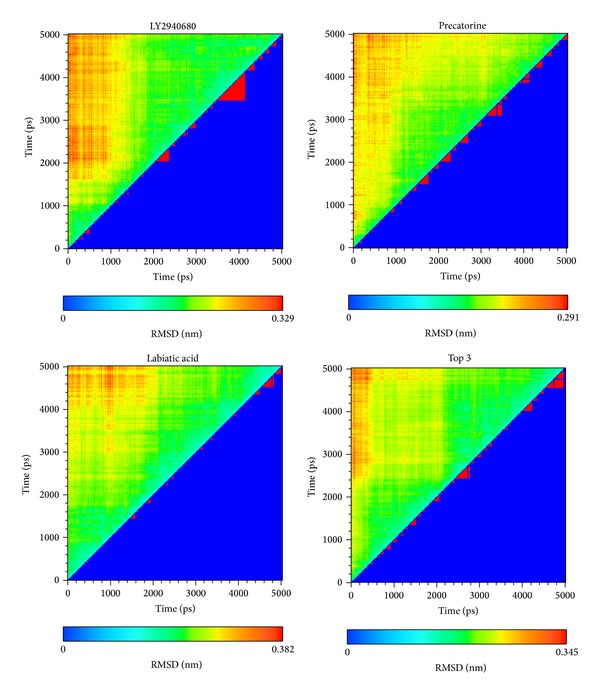
Root-mean-square deviation value (upper left half) and graphical depiction of the clusters with cutoff 0.105 nm (lower right half) for Smo protein complexes with LY2940680, precatorine, labiatic acid, and 2,2′-[benzene-1,4-diylbis(methanediyloxybenzene-4,1-diyl)]bis(oxoacetic acid).

**Figure 12:**
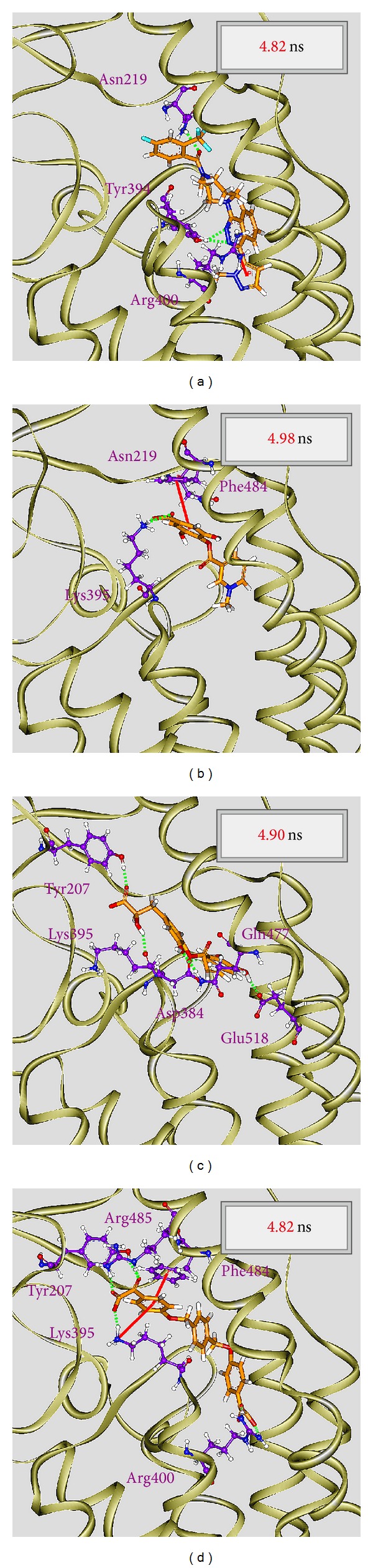
Docking poses of middle RMSD structure in the major cluster for Smo protein complexes with LY2940680, precatorine, labiatic acid, and 2,2′-[benzene-1,4-diylbis(methanediyloxybenzene-4,1-diyl)]bis(oxoacetic acid).

For the MD simulation, the representative structures of each complex under dynamic conditions were identified by the cluster analysis with a RMSD cutoff of 0.105 nm. According to the RMSD values and graphical depiction of the clusters for Smo protein complexes with LY2940680, precatorine, labiatic acid, and 2,2′-[benzene-1,4-diylbis(methanediyloxybenzene-4,1-diyl)]bis(oxoacetic acid) illustrated in Figure 11, the docking poses of the representative structures for Smo protein complex with LY2940680 and the top three TCM compounds were illustrated in Figure 12. For LY2940680, there exist the stable H-bonds with residues Asn219 and Arg400 under dynamic conditions. In addition, it forms an H-bond with Tyr394 after MD simulation. Precatorine has stable *π* interactions with residue Phe484 and H-bonds with Lys395. After MD simulation, it forms an H-bond with residue Asn219. Labiatic acid has stable H-bonds with residues Tyr207, Lys395 and forms the H-bonds with residues Asp384, Gln477, and Glu518. The top 3 TCM compounds have stable *π* interactions with residue Phe484 and H-bonds with residue Lys395. Moreover, the interaction with residue Arg400 was changed from *π* interaction to H-bond and forms the H-bonds with residues Tyr207 and Arg285 after MD simulation.

**Figure 13:**
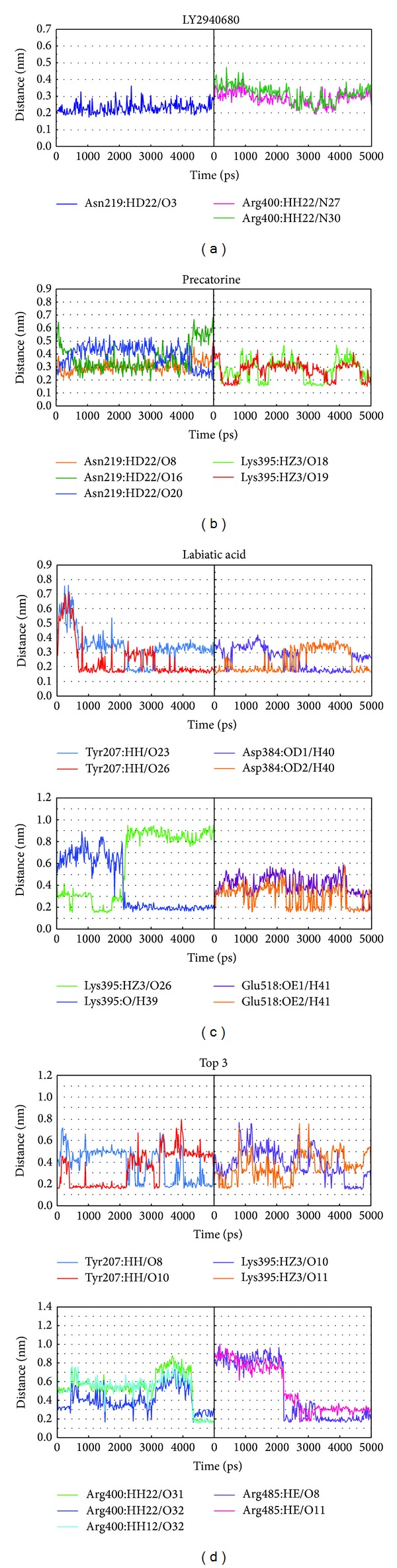
Distances of hydrogen bonds with common residues during 5000 ps of MD simulation.

To analyze the variation of H-bonds for key residues in each protein-ligand complex, the H-bond occupancy for key residues of Smo protein with top three candidates and LY2940680 overall 5000 ps of MD simulation was listed in Table 1, and the distance variations of each H-bond were displayed in Figure 13. They indicate that the H-bonds between LY2940680 and residues Asn219, Tyr394, Arg400 were stabilized over 5000 ps of MD simulation. In addition, the H-bonds between top three TCM compounds and residues mentioned above were also stabilized. Comparing to docking poses between docking simulation (Figure 4) and MD simulation (Figure 12), LY2940680 and the top three TCM compounds maintain most of interactions with Smo protein, which keep the ligand binding stable in the binding domain.

## 4. Conclusion

This study aims to investigate the potent TCM candidates for Smo protein. The top three TCM compounds, precatorine, labiatic acid, and 2,2′-[benzene-1,4-diylbis(methanediyloxybenzene-4,1-diyl)]bis(oxoacetic acid), have displayed higher potent binding affinities than the positive control, LY2940680, in the docking simulation. The docking poses of top three TCM compounds have similar *π* interaction with residue Phe484 and interaction with common residues Lys395 and Arg400. The MD simulations are employed to optimize the result of docking simulation and validate the stability of H-bonds between each ligand and Smo protein under dynamic conditions. For the MD simulation, the top three TCM compounds maintain most of interactions with Smo protein, which keep the ligand binding stable in the binding domain. Hence, we propose precatorine, labiatic acid, and 2,2′-[benzene-1,4-diylbis(methanediyloxybenzene-4,1-diyl)]bis(oxoacetic acid) as potential lead compounds for further study in drug development process with the Smo protein.

## Figures and Tables

**Table 1:** H-bond occupancy for key residues of Smo protein with top three candidates and LY2940680 overall 5000 ps of molecular dynamics simulation.

Name	H-bond interaction	Occupancy
LY2940680	Asn219:HD22/O3	96%
	Tyr394:HH/N27	80%
	Tyr394:HH/N30	80%
	Arg400:HH22/N27	61%
	Arg400:HH22/N30	33%

Precatorine	Asn219:HD22/O8	52%
	Asn219:HD22/O16	29%
	Asn219:HD22/O20	18%
	Asp384:OD1/H29	46%
	Lys395:HZ3/O18	55%
	Lys395:HZ3/O19	57%

Labiatic acid	Tyr207:HH/O21	7%
	Tyr207:HH/O23	23%
	Tyr207:HH/O26	80%
	Asn219:HD22/O24	17%
	Asp384:OD1/H40	65%
	Asp384:OD2/H40	66%
	Lys395:O/H30	9%
	Lys395:O/H39	57%
	Lys395:HZ3/O23	9%
	Lys395:HZ3/O26	24%
	Glu518:OE1/H41	5%
	Glu518:OE2/H41	40%

Top 3	Tyr207:HH/O8	34%
	Tyr207:HH/O10	43%
	Tyr207:HH/O11	8%
	Tyr394:HH/O29	2%
	Tyr394:HH/O31	73%
	Lys395:HZ3/O8	10%
	Lys395:HZ3/O10	23%
	Lys395:HZ3/O11	27%
	Arg400:HH12/O32	14%
	Arg400:HH22/O31	14%
	Arg400:HH22/O32	18%
	Arg485:HH22/O11	2%
	Arg485:HE/O8	48%
	Arg485:HE/O10	10%
	Arg485:HE/O11	25%

H-bond occupancy cutoff: 0.3 nm.

Top 3: 2,2′-[benzene-1,4-diylbis(methanediyloxybenzene-4,1-diyl)]bis(oxoacetic acid).
